# Ten-year re-validation of the fracture risk assessment tool (FRAX®) in Taiwan

**DOI:** 10.1007/s11657-026-01745-2

**Published:** 2026-07-21

**Authors:** Chia-Chun Li, I.‑Ting Liu, Fu‑Wen Liang, Yin-Fan Chang, Chin-Sung Chang, Zih-Jie Sun, Shau-Huai Fu, Chung-Yi Li, Li-Chieh Kuo, Chih-Hsing Wu

**Affiliations:** 1 Institute of Allied Health Sciences, College of Medicine, National Cheng Kung University, Tainan, Taiwan; 2 Department of Family Medicine, College of Medicine, National Cheng Kung University, Tainan, Taiwan; 3 Department of Family Medicine, E-DA Hospital, Kaohsiung, Taiwan; 4 Department of Geriatric Medicine, E-DA Hospital, Kaohsiung, Taiwan; 5 School of Medicine, College of Medicine, I-Shou University, Kaohsiung, Taiwan; 6 Department of Public Health, College of Health Science, Kaohsiung Medical University, Kaohsiung, Taiwan; 7 Department of Medical Research, Kaohsiung Medical University Hospital, Kaohsiung, Taiwan; 8 Department of Family Medicine, College of Medicine, National Cheng Kung University Hospital, National Cheng Kung University, Tainan, Taiwan; 9 Department of Family Medicine, Douliu Branch, College of Medicine, National Cheng Kung University Hospital, National Cheng Kung University, Yunlin, Taiwan; 10 Department of Orthopedics, National Taiwan University Hospital Yun-Lin Branch, Douliu, Taiwan; 11 Department of Orthopedic Surgery, National Taiwan University Hospital, Taipei, Taiwan; 12 Department of Public Health, College of Medicine, National Cheng Kung University, Tainan, Taiwan; 13 Department of Public Health, College of Public Health, China Medical University, Taichung, Taiwan; 14 Department of Healthcare Administration, College of Medical and Health Science, Asia University, Taichung, Taiwan; 15 Department of Occupational Therapy, College of Medicine, National Cheng Kung University, Tainan, Taiwan; 16 Institute of Gerontology, Medical College, National Cheng Kung University, Tainan, Taiwan

**Keywords:** FRAX, Data-driven threshold, Fracture risk prediction, Bone mineral density, Population-specific, Osteoporotic fracture

## Abstract

**Summary:**

The Taiwan-specific fracture risk assessment tool (FRAX) model is widely used to estimate 10-year fracture risk. However, FRAX with bone mineral density (BMD) may underestimate fracture risk, particularly in older adults.

**Purpose:**

To evaluate the predictive performance and calibration of the Taiwan-specific FRAX calculator using complete 10-year real-world outcomes and to establish data-driven, population-specific screening thresholds.

**Methods:**

A total of 1,976 participants aged ≥ 40 years were recruited from two population-based cohorts in Taiwan (2009–2010) and followed for 10 years via linkage with the National Health Insurance Research Database. Ten-year probabilities of major osteoporotic fracture (MOF) and hip fracture (HF) were estimated using Taiwan-specific FRAX calculators. Model calibration was evaluated using observed-to-expected (O/E) ratios. Statistically optimal thresholds were determined via receiver operating characteristic analysis maximizing the Youden index.

**Results:**

During follow-up, 213 (10.8%) participants experienced MOF, and 93 (4.7%) experienced HF. FRAX without BMD demonstrated strong calibration for both MOF (O/E = 1.08; 95% CI: 0.94–1.24) and HF (O/E = 1.10; 95% CI: 0.89–1.34). Conversely, FRAX with BMD systematically underestimated risk, particularly among individuals aged ≥ 65 years (MOF O/E = 1.23; 95% CI: 1.06–1.42). Age, female sex, prior fracture, and low BMD were significant independent risk factors. Crucially, population-specific data-driven thresholds were identified via ROC analysis at 9.0% for MOF and 4.5% for HF using FRAX without BMD, and 8.5% for MOF and 4.0% for HF using FRAX with BMD, respectively.

**Conclusion:**

FRAX**®** remains a useful and widely applied tool for fracture risk prediction in Taiwan. However, fracture risk may be underestimated, particularly in adults aged ≥ 65 years. Taiwan-specific, data-driven thresholds may improve risk stratification and support more targeted fracture prevention strategies.

**Supplementary Information:**

The online version contains supplementary material available at 10.1007/s11657-026-01745-2.

## Introduction

Osteoporotic fractures pose a growing public health challenge in aging populations [1]. Fragility fractures of the hip, spine, and forearm are associated with substantial morbidity, mortality, and healthcare costs, with their incidence continuing to rise worldwide [2, 3]. Low bone mineral density (BMD) contributes significantly to this burden, accounting for millions of disability-adjusted life years and hundreds of thousands of deaths annually [4]. Despite the availability of effective diagnostic and pharmacologic interventions, osteoporosis remains under-recognized and under-treated, frequently presenting clinically only after an initial fragility fracture has occurred [5, 6].

As an insidious and largely asymptomatic condition [7], osteoporosis requires robust fracture risk assessment tools to enable accurate risk stratification and guide targeted preventive strategies [8, 9]. In Taiwan, although the age-standardized prevalence and incidence of osteoporosis declined between 2008 and 2019, the overall disease burden remains considerable due to rapid population aging; furthermore, persistently high post-fracture mortality highlights ongoing gaps in secondary prevention [10]. Consequently, osteoporosis management in Taiwan has shifted toward a comprehensive, risk-based approach that emphasizes long-term, sequential pharmacologic therapy integrated with fracture liaison services and lifestyle interventions to optimize prevention and improve treatment adherence [11].

The FRAX tool is widely regarded as the global standard for estimating 10-year osteoporotic fracture probability based on clinical risk factors, with or without femoral neck BMD. Accurately identifying high-risk individuals before a fracture occurs can critically inform clinical decisions and reduce subsequent fracture incidence, particularly among postmenopausal women and older adults [12]. Although FRAX has been validated across numerous regions with nearly 100 country-specific models currently available [13, 14], further validation within localized populations is essential. Developing ethnic-specific fracture risk prediction tools and establishing country-specific screening thresholds grounded in local epidemiological data remain imperative for optimal clinical utility [15, 16].

Moreover, because FRAX estimates a 10-year fracture probability, rigorous validation requires longitudinal cohort data with a full decade of follow-up. In 2022, our research team confirmed the favorable performance of FRAX in Taiwan using cohort data with a mean follow-up of 6.8 years [17]. To enhance the precision of this assessment, we extended the follow-up of these community-based cohorts to a complete 10-year period. Accordingly, the present study addresses a critical methodological gap in prior regional validation studies by directly evaluating 10-year FRAX probabilities against observed 10-year real-world outcomes. Furthermore, we aim to determine the optimal Taiwan-specific treatment thresholds and analyze key clinical risk factors to elucidate their relative contributions to fracture risk within this population.

## Methods

### Participants

Participants aged ≥ 40 years were recruited from two population-based cohorts in Taiwan between 2009 and 2010, with protocol approval obtained from the Institutional Review Board of National Cheng Kung University Hospital (IRB Nos. ER-98–125, ER-99–111, and ER-98–084) [18–20]. The first cohort, recruited in 2010 from Yunlin County in central Taiwan, comprised 524 men and 676 women with a mean age of 59.6 ± 11.4 years [20]. The second cohort, recruited from Tianliao Township in southern Taiwan, included 408 men and 368 women with a mean age of 74.2 ± 6.1 years [18, 19, 21, 22]. Exclusion criteria were incomplete baseline FRAX variable data or the inability to undergo dual-energy X-ray absorptiometry (DXA) assessment. Detailed recruitment procedures, response rates, and eligibility criteria have been described previously [18–22]. No statistically significant differences in mean age or age distribution were observed between respondents and nonrespondents in either cohort. The methodological compatibility and validity of integrating these two cohorts have been established in previous peer-reviewed studies [17, 23, 24].

### Measurements

Structured questionnaires were administered at baseline by trained nurses to collect information on all clinical risk factors (CRFs) incorporated into the FRAX algorithm, with definitions aligning primarily with the original FRAX framework. To minimize recall bias, data regarding rheumatoid arthritis were obtained from medical chart records. Height and weight were measured following an overnight fast [19]. BMD at the femoral neck, total hip, and lumbar spine was assessed using DXA via an Explorer Hologic system in Tianliao and a Prodigy GE Lunar system in Yunlin [17, 25]. All densitometric procedures adhered to the International Society for Clinical Densitometry (ISCD) standards [23, 26], including routine calibration and precision monitoring. T-scores were calculated using the manufacturer’s reference database. Ten-year probabilities for major osteoporotic fractures (MOF; comprising hip, clinical spine, forearm, and proximal humerus fractures) and hip fractures (HF) were estimated using the Taiwan-specific FRAX calculator, calculated both with and without the incorporation of femoral neck BMD [27]. The Taiwan-specific FRAX calculator was developed by the team of Professor John A. Kanis using the standard FRAX methodology in 2010. The Taiwan age- and sex-specific hip fracture incidence rates in 1996–2002 were used [28] and corresponding Taiwan age-specific mortality data were incorporated into the model. As in many other country-specific FRAX models, the age- and sex-specific incidence of non-hip MOF was imputed from hip fracture incidence.

To evaluate 10-year predictive performance, participants were linked to Taiwan’s National Health Insurance Research Database (NHIRD), which provides comprehensive longitudinal electronic health records covering more than 99.9% of the population [29]. To guarantee a complete 10-year follow-up, the study endpoint was defined as exactly 10 years after each participant’s index baseline date. Incident hip fractures were identified using validated International Classification of Diseases, Ninth and Tenth Revision, Clinical Modification (ICD-9-CM and ICD-10-CM) codes within NHIRD inpatient claims, consistent with established epidemiological algorithms [30]. Major osteoporotic fractures (MOF), including clinical spine, forearm, and proximal humerus fractures, were identified using corresponding inpatient ICD-9/10-CM diagnosis codes based on established epidemiological definitions (Supplemental Table S1), although formal validation studies for these non-hip fracture algorithms in the NHIRD are currently unavailable. Mortality data were retrieved from the National Death Registry. No participants were lost to follow-up for non-death reasons.

To minimize potential confounding from anti-osteoporosis medications (AOMs), therapies were tracked using the World Health Organization (WHO) Anatomical Therapeutic Chemical (ATC) classification system. Evaluated AOMs included raloxifene, bazedoxifene, alendronate, risedronate, ibandronate, denosumab, zoledronic acid, teriparatide, and romosozumab. Individuals receiving these agents for non-osteoporotic indications (e.g., malignancy or Paget's disease) were excluded. AOM users were defined as participants with at least one outpatient prescription record for any approved AOM during the 10-year follow-up; those without such records were classified as non-users.

### Statistical analysis

Continuous variables are expressed as means ± standard deviations and compared using Student's *t*-tests, while categorical variables are reported as frequencies with percentages and compared using Chi-square tests.

To mitigate common methodological pitfalls in external validation, multiple statistical dimensions were evaluated. Calibration of the 10-year FRAX probabilities was assessed by calculating observed-to-expected (O/E) ratios alongside their corresponding 95% confidence intervals (CIs) and *p* values. Expected fracture counts were determined by summing individual-level FRAX probabilities, whereas observed fractures represented actual incident events during the 10-year follow-up. The 95% CIs for O/E ratios were derived assuming a Poisson distribution for the observed counts. No strict a priori O/E threshold was predefined; calibration was interpreted based on proximity to 1.0, the width of the 95% confidence intervals, and clinical relevance. Calibration across risk spectrums was further analyzed by comparing observed and predicted fracture probabilities across risk quintiles.

Although FRAX inherently accounts for mortality as a competing risk during probability calculation, competing-risk regression analyses were additionally performed to interpret the specific fracture predictors within this aging cohort. Fine and Gray’s subdistribution hazard regression model was applied to estimate subdistribution hazard ratios (sHRs) and 95% CIs for each CRF included in the FRAX algorithm (with and without BMD) [31]. Mortality, verified via the national registry, was treated as the competing risk event [32]. To isolate the potential impact of anti-osteoporosis therapy on fracture incidence, a sensitivity analysis stratified by AOMs use was conducted.

Statistically optimal screening thresholds were identified using receiver operating characteristic (ROC) curve analysis by maximizing Youden’s index. Sensitivity and specificity were evaluated across a range of potential thresholds for models with and without BMD. These ROC-derived thresholds are intended to optimize discriminatory performance and do not represent formal cost-effectiveness-based or policy-level intervention thresholds. All statistical tests were two-sided, and a *p* value < 0.05 was considered statistically significant. Statistical analyses were performed using SAS software version 9.4 (SAS Institute Inc., Cary, NC, USA).

## Results

A total of 1,976 participants were included in the analysis. During the 10-year follow-up period, 213 (10.8%) participants experienced MOF and 93 (4.7%) experienced HF during the 10-year follow-up period. As shown in Table 1, participants who sustained an MOF were significantly older than those who remained fracture-free (71.7 ± 10.1 years vs. 64.6 ± 12.1 years; *p* < 0.001). The proportion of women was also significantly higher in the MOF group than in the non-fracture group (64.8% vs. 51.4%; p = 0.002). Based on fracture event–driven tracking, the median follow-up duration was 10 years (IQR: 0 years) for participants without MOF and 10 years (IQR: 2 years) for those with MOF. The all-cause mortality rate was significantly elevated in the MOF group compared to the non-fracture group (34.7% vs. 16.3%; *p* < 0.001). Utilizing an MOF probability threshold of ≥ 10%, 31.8% of participants evaluated with BMD and 35.3% of those evaluated without BMD were classified as moderate-to-high risk, capturing 59.6% and 63.4% of subsequent fractures, respectively. At a higher threshold of ≥ 20%, the proportion categorized as high risk decreased to 8.1% (with BMD) and 10.1% (without BMD), accounting for 22.1% and 24.9% of total fracture events.

**Table 1:** Baseline characteristics of study participants

Variables	Overall (n = 1976)	Without MOF (n = 1763)	With MOF (n = 213)	*p* value
Sex				0.0002
Male (%)	932(47.2)	857(48.6)	75(35.2)	
Female (%)	1044(52.8)	906(51.4)	138(64.8)	
Age, years	65.4 ± 12.1	64.6 ± 12.1	71.7 ± 10.1	< 0.0001
< 65 years old	784(39.7)	749(42.5)	35(16.4)	< 0.0001
> = 65 years old	1192(60.3)	1014(57.5)	178(83.6)	
Weight, kg	61.1 ± 10.9	61.5 ± 10.8	57.7 ± 10.4	< 0.0001
Height, cm	157.4 ± 8.4	157.8 ± 8.2	154.0 ± 8.8	< 0.0001
BMI, kg/m^2^	24.6 ± 3.6	24.6 ± 3.6	24.2 ± 3.3	0.13
Prior falls	399(20.2)	336(19.1)	63(29.6)	0.0003
Femoral neck T-score, SD	−1.3 ± 1.3	−1.2 ± 1.3	−2.2 ± 1.2	< 0.0001
Median	−1.4	−1.3	−2.3	
IQR	1.7	1.7	1.5	
Duration of follow-up, years	9.3 ± 1.8	9.4 ± 1.8	9.0 ± 1.9	0.0030
Median	10	10	10	
IQR	0	0	2	
AOMs users	102(5.2)	20(1.1)	82(38.5)	< 0.0001
Death	362(18.3)	288(16.3)	74(34.7)	< 0.0001
FRAX CRFs				
Previous Fracture	200(10.1)	159(9.0)	41(19.3)	< 0.0001
Parent Fractured Hip	119(6.0)	110(6.2)	9(4.2)	0.24
Current Smoking	280(14.2)	255(14.5)	25(11.7)	0.28
Alcohol	204(10.3)	189(10.7)	15(7.0)	0.10
Glucocorticoids	42(2.1)	36(2.0)	6(2.8)	0.46
Rheumatoid arthritis history	17(0.9)	13(0.7)	4(1.9)	0.09
Secondary osteoporosis	61(3.1)	52(2.9)	9(4.2)	0.31
FRAX MOF				< 0.0001
with BMD	9.0 ± 7.7	8.4 ± 7.1	14.4 ± 10.0	
< 10	1348(68.2)	1262(71.6)	86(40.4)	
> = 10 and < 20	469(23.7)	389(22.1)	80(37.6)	
> = 20	159(8.1)	112(6.4)	47(22.1)	
without BMD	10.0 ± 7.9	9.3 ± 7.2	15.6 ± 10.6	
< 10	1278(64.7)	1200(68.1)	78(36.6)	
> = 10 and < 20	499(25.3)	417(23.7)	82(38.5)	
> = 20	199(10.1)	146(8.3)	53(24.9)	
FRAX HF				< 0.0001
with BMD	3.6 ± 4.7	3.2 ± 4.1	6.9 ± 7.1	
without BMD	4.3 ± 5.0	3.8 ± 4.3	8.1 ± 8.3	

χ^2^ test for categorical variables, data are expressed as numbers (%); *t* test for continuous variables, data are expressed as means ± standard deviation

MOF, major osteoporotic fracture; HF, hip fracture; BMI, body mass index; FRAX, fracture risk assessment tool; CRFs, clinical risk factors; BMD, bone mineral density

When applying the FRAX model without BMD, the algorithm demonstrated generally strong calibration, with O/E ratios approaching 1.0 for both MOF (O/E = 1.08; 95% CI: 0.94–1.24) and HF (O/E = 1.10; 95% CI: 0.89–1.34) (Table 2). Age-stratified analyses revealed that among participants < 65 years (Supplemental Table S2), the model without BMD maintained a close alignment between observed and predicted risks, with no statistically significant differences identified (MOF O/E = 0.81; 95% CI: 0.58–1.12).

**Table 2:** Calibration of FRAX-predicted 10-year probabilities of major osteoporotic and hip fractures

	Observed fracture	Expected fracture	O/E	95% CI
FRAX with BMD				
MOF	213	178.07	1.20	1.04–1.37*
HF	93	70.91	1.31	1.07–1.60*
FRAX without BMD				
MOF	213	196.68	1.08	0.94–1.24
HF	93	84.97	1.10	0.89–1.34

FRAX, fracture risk assessment tool; BMD, bone mineral density; MOF, major osteoporotic fracture; HF, hip fracture. * *p* < 0.05

Conversely, the FRAX model with BMD systematically and substantially underestimated fracture probabilities. The overall O/E ratio for the 10-year MOF risk was 1.20 (95% CI: 1.04–1.37), and a comparable underestimation pattern was observed for HF (O/E = 1.31; 95% CI: 1.07–1.60) (Table 2). This underestimation was most pronounced among participants aged ≥ 65 years, where the BMD-input model underestimated both MOF (O/E = 1.23; 95% CI: 1.06–1.42) and HF (O/E = 1.40; 95% CI: 1.14–1.72). Calibration analysis across predicted risk quintiles revealed a consistent trend of miscalibration: the lower-risk quintiles exhibited an overestimation of fracture risk, whereas the higher-risk quintiles demonstrated underestimation. This trend was consistent across all models but appeared more pronounced in those incorporating BMD (Supplemental Table S3).

Multivariate competing-risk regression models indicated that older age, female sex, and a history of prior fracture were independently associated with increased risks of both MOF and HF (Table 3). Each 1-year increase in age was associated with a 6% increase in MOF risk (sHR = 1.06; 95% CI: 1.05–1.08) and a 13% increase in HF risk (sHR = 1.13; 95% CI: 1.10–1.15). Women exhibited a more than twofold higher risk of sustaining an MOF compared to men (sHR = 2.15; 95% CI: 1.54–3.01). Furthermore, a history of prior fracture nearly doubled the risk of both subsequent MOF (sHR = 1.93; 95% CI: 1.36–2.75) and HF (sHR = 1.93; 95% CI: 1.11–3.35). As detailed in Supplemental Table S4, each 1 standard deviation increase in femoral neck T-score was associated with a 33% reduction in MOF risk (sHR = 0.67; 95% CI: 0.59–0.77) and a 34% reduction in HF risk (sHR = 0.66; 95% CI: 0.53–0.81). Other baseline CRFs, including current smoking, glucocorticoid use, rheumatoid arthritis, and high alcohol intake, did not reach statistical significance after multivariate adjustment in this cohort.

**Table 3:** Cox proportional hazard model with competing risk of MOF and HF for FRAX without BMD

FRAX CRFs	MOF		HF	
	Sub-distribution HR	*p* value	Sub-distribution HR	*p* value
Female	2.15(1.54–3.01)	< 0.0001	1.61(0.98–2.62)	0.06
Age, year	1.06(1.05–1.08)	< 0.0001	1.13(1.10–1.15)	< 0.0001
BMI, kg/m^2^	0.98(0.95–1.02)	0.26	0.95(0.90–1.01)	0.11
Previous Fracture	1.93(1.36–2.75)	0.0003	1.93(1.11–3.35)	0.02
Parent Fractured Hip	0.83(0.43–1.61)	0.59	0.90(0.28–2.85)	0.85
Current Smoking	1.47(0.93–2.32)	0.10	1.72(0.91–3.24)	0.10
Alcohol	1.19(0.68–2.09)	0.54	1.14(0.49–2.67)	0.76
Glucocorticoids	1.06(0.49–2.31)	0.88	1.82(0.67–4.98)	0.24
Rheumatoid arthritis history	2.00(0.75–5.33)	0.17	3.48(0.88–13.73)	0.08
Secondary osteoporosis	0.99(0.49–2.00)	0.98	0.86(0.26–2.81)	0.80

FRAX, fracture risk assessment tool; MOF, major osteoporotic fracture; HF, hip fracture; BMD, bone mineral density; CRFs, clinical risk factors; HR, hazard ratio; BMI, body mass index

When stratified by AOMs use during follow-up, core risk relationships were largely preserved. Age and secondary osteoporosis remained significantly associated with MOF risk among AOMs users (Supplemental Table S5). In the non- AOMs cohort, age, a history of rheumatoid arthritis, and prior fracture emerged as key determinants of MOF risk, whereas BMI served as a significant protective factor against HF (Supplemental Table S6). Although secondary osteoporosis among AOMs users and rheumatoid arthritis among non-users achieved statistical significance in these stratified models, their 95% CIs overlapped widely, necessitating a cautious interpretation of these subgroup variations.

**Fig. 1:**
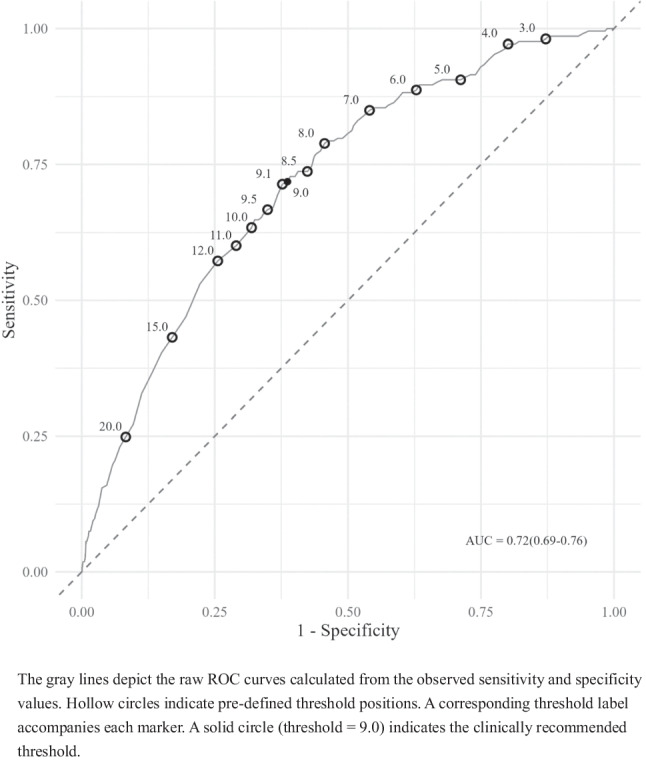
The FRAX without BMD threshold for predicting major osteoporotic fracture

**Fig. 2:**
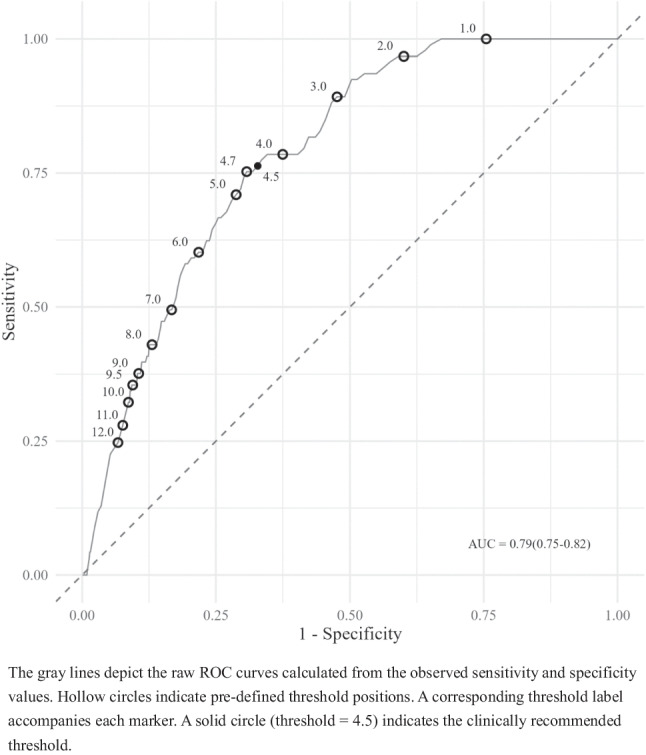
The FRAX without BMD threshold for predicting hip fracture

ROC curve analyses were performed to determine statistically optimal thresholds for the FRAX model without BMD (Table 4, Figs. 1 and 2). For MOF, the threshold that maximized the Youden index was a 10-year probability of 9.1%, yielding a sensitivity of 0.71, a specificity of 0.62, a maximum Youden index of 0.33, and an area under the curve (AUC) of 0.72. For HF, the optimal threshold was identified at 4.7%, which provided a sensitivity of 0.75, a specificity of 0.69, a Youden index of 0.45, and an AUC of 0.79. ROC curve analyses were also performed for the FRAX model with BMD (Supplemental Table S7 and Supplemental Figures S1 and S2). The statistically optimal thresholds identified by the highest Youden index were 8.2% for MOF and 3.8% for HF. Considering diagnostic performance and clinical applicability, thresholds of 8.5% for MOF and 4.0% for HF were selected as the recommended data-driven thresholds. The AUCs were 0.72 for MOF and 0.81 for HF. Overall, the optimal FRAX screening thresholds generated with and without BMD demonstrated remarkably similar distributions and diagnostic performance.

**Table 4:** The FRAX without BMD threshold for predicting MOF and HF

MOF				HF			
Threshold (%)	Sensitivity	Specificity	Youden	Threshold (%)	Sensitivity	Specificity	Youden
20.0^a^	0.25	0.92	0.17	12.0	0.25	0.93	0.18
15.0^c^	0.43	0.83	0.26	11.0	0.28	0.92	0.20
12.0	0.57	0.74	0.32	10.0	0.32	0.91	0.24
11.0	0.60	0.71	0.31	9.5	0.35	0.91	0.26
10.0	0.63	0.68	0.31	9.0	0.38	0.89	0.27
9.5^b^	0.67	0.65	0.32	8.0	0.43	0.87	0.30
9.1^d^	0.71	0.62	0.34	7.0 ^c^	0.49	0.83	0.33
9.0	0.72	0.61	0.33	6.0 ^c^	0.60	0.78	0.38
8.5	0.74	0.58	0.31	5.0	0.71	0.71	0.42
8.0	0.79	0.54	0.33	4.7 ^d^	0.75	0.69	0.45
7.0	0.85	0.46	0.31	4.5	0.76	0.67	0.44
6.0	0.89	0.37	0.26	4.0 ^b^	0.78	0.63	0.41
5.0	0.91	0.29	0.19	3.0 ^a^	0.89	0.52	0.42
4.0	0.97	0.20	0.17	2.0	0.97	0.40	0.37
3.0	0.98	0.13	0.11	1.0	1.00	0.25	0.25

FRAX, fracture risk assessment tool; BMD, bone mineral density; MOF, major osteoporotic fracture; HF, hip fracture

a. NOF threshold [39, 40], b. Liu’s threshold [17], c. Chan’s threshold [41], d. this study suggested threshold with highest Youden index

## Discussion

In this population-based cohort with a complete 10-year follow-up, the FRAX model incorporating BMD systematically underestimated fracture risk, particularly among adults aged ≥ 65 years, whereas the model without BMD demonstrated superior overall calibration. The data-driven thresholds derived from the model without BMD (9.0% for MOF and 4.5% for HF) provided balanced diagnostic performance, suggesting higher clinical utility for fracture risk stratification in Taiwanese populations. By directly validating predicted 10-year probabilities against observed 10-year fracture outcomes, this study addresses a major methodological limitation of prior regional validation studies.

Consistent with previous investigations, older age, female sex, prior fracture history, and low femoral neck BMD emerged as the paramount predictors of both MOF and HF in this cohort [25, 33–35]. Notably, the direction of calibration error observed herein contrasts with reports from several Western cohorts, where FRAX tended to overestimate fracture risk in Asian populations [36]. Conversely, our findings demonstrate a persistent underestimation of fracture probability when utilizing the Taiwan FRAX calculator with BMD, especially among older adults. This underestimation highlights the intrinsic complexity of fracture risk modeling in aging populations, reflecting the multifactorial contributions of geriatric syndromes such as falls, frailty, and a high comorbidity burden. Although slightly different statistical thresholds were observed between the models with and without BMD, these should not be interpreted as separate clinical pathways. In clinical practice, FRAX without BMD may serve as an effective first-line community screening tool, whereas FRAX with BMD can refine risk estimation following densitometric assessment.

Several factors may explain why the BMD-integrated FRAX model underestimates fracture risk in Taiwan. First, secular increases in life expectancy and age-related frailty may result in a higher real-world fracture incidence than assumed in the original FRAX reference datasets. Second, discrepancies between measured BMD and true skeletal fragility may arise from variations in ethnic reference standards or densitometer calibration [37–39]. Third, competing risks—particularly all-cause mortality among older adults—differentially influence probability estimates when BMD is embedded. Fourth, because fracture ascertainment relied on inpatient claims, non-hospitalized or asymptomatic vertebral fractures may have been under-identified, partially explaining the high proportion of HFs within the recorded MOF events. Crucially, however, HFs in Taiwan almost uniformly require hospitalization, minimizing under-ascertainment for this specific outcome. However, these findings suggest potential miscalibration, particularly in older adults, and warrant further assessment of calibration in larger independent Taiwanese cohorts.

Our results align with data from other East Asian regions, including Japan, Korea, and Hong Kong, where FRAX demonstrates acceptable discrimination but frequently requires localized calibration to achieve optimal concordance [13]. The closer calibration observed for the model without BMD in our study suggests that clinical risk factors (CRFs) alone may effectively capture population-level fracture risks in Taiwan. Accordingly, parallel risk assessments using tools like the Osteoporosis Self-Assessment Tool for Taiwan women (OSTAi) or men (MOSTAi) [23] may enhance community-level screening accuracy. These findings do not diminish the clinical value of DXA for individual diagnosis, but rather highlight the limitations of directly embedding baseline BMD into population-level risk models without addressing the algorithm's inherent structural constraints. Specifically, FRAX excludes critical risk contributors such as fall frequency [24], frailty, and age-related deterioration in bone microarchitecture, which are not fully captured by DXA-derived BMD.

The data-driven thresholds identified herein were lower than both the global reference thresholds recommended by the National Osteoporosis Foundation [40, 41] and the cost-effectiveness-based thresholds proposed by Chan et al. [42]. Compared to the thresholds reported by Liu et al. [17], our ROC-derived values demonstrated superior Youden indices, indicating an optimized balance of sensitivity and specificity. Crucially, by leveraging complete 10-year fracture outcomes, this study enhances the longitudinal validity and real-world applicability of the proposed thresholds, providing compelling empirical support for the population-specific recalibration of FRAX thresholds in Taiwan rather than relying solely on global or economic benchmarks.

In application, our proposed threshold ranges (9.0% to 9.5% for MOF and 4.0% to 4.5% for HF) are intended to guide community screening and primary care by identifying high-risk individuals for targeted DXA referrals. However, the relatively low positive predictive value indicates that many individuals categorized as high risk will not sustain a fracture within a decade, underscoring the necessity of integrating FRAX scores with clinical judgment, fall risk assessments, and shared decision-making. We emphasize that these are not final intervention thresholds; formal clinical thresholds must be established only after comprehensive evaluation of diagnostic criteria, insurance reimbursement policies, willingness to pay, and national healthcare priorities, etc.

This study possesses notable strengths, including its population-based design, complete 10-year follow-up, and the application of competing-risk models to rigorously account for mortality. Linkage with the NHIRD enabled robust ascertainment of longitudinal fracture outcomes, strengthening the validity of our calibration assessment.

Nonetheless, several limitations warrant consideration. First, high rates of AOM use during follow-up, particularly among individuals who sustained fractures, introduce potential confounding from treatment-related risk reductions. However, our treatment-stratified sensitivity analyses demonstrated that the predictive significance of core CRFs remained robust. Second, although population-based, this cohort may not fully represent older adults with limited healthcare access. Third, the relatively small number of incident HF cases, though consistent with regional epidemiological trends, limits the statistical power and precision of certain subgroup analyses. Finally, while this cohort provides invaluable long-term data, the absence of a parallel, independent Taiwanese replication cohort suggests that these findings should be further confirmed in larger datasets before formal model recalibration is implemented.

In summary, FRAX® remains a widely adopted tool for fracture risk assessment in Taiwan. The identified population-specific thresholds provide an optimized balance of sensitivity and specificity, supporting robust risk stratification. Age, sex, prior fracture history, and low femoral neck BMD persist as key independent predictors of skeletal events. Integrating these customized reference thresholds into multi-step, evidence-based clinical pathways will facilitate more effective osteoporosis prevention and fracture risk management in Taiwan.

## Supplementary Information

Below is the link to the electronic supplementary material.

Supplementary file1 (DOCX 202 KB)

## Data Availability

The data supporting this study's findings are not publicly available because they were obtained from the Taiwan National Health Insurance Research Database (NHIRD), which is subject to data protection regulations and licensing restrictions. Consequently, the authors are not permitted to share the raw data. Aggregated data, analytical methods, and additional information related to the analyses are available from the corresponding author upon reasonable request.

## References

[1] NIH Consensus Development Panel on Osteoporosis Prevention, D Therapy (2001) Osteoporosis prevention, diagnosis, and therapy. JAMA 285:785–795 11176917 10.1001/jama.285.6.785

[2] Sözen T, Özışık L, Başaran N (2017) An overview and management of osteoporosis. Eur J Rheumatol 4:46–56 28293453 10.5152/eurjrheum.2016.048 PMC5335887

[3] Clynes MA, Harvey NC, Curtis EM et al (2020) The epidemiology of osteoporosis. Br Med Bull 133:105–117 32282039 10.1093/bmb/ldaa005 PMC7115830

[4] Hsieh E, Bryazka D, Ong KL et al (2025) The global, regional, and national burden attributable to low bone mineral density, 1990–2020: an analysis of a modifiable risk factor from the Global Burden of Disease Study 2021. Lancet Rheumatol 7:e873–e894 40972625 10.1016/S2665-9913(25)00105-5 PMC12623303

[5] Kanis JA on behalf of the World Health Organization Scientific Group (2007) Assessment of osteoporosis at the primary health-care level. Technical Report. World Health Organization Collaborating Centre for metabolic bone diseases, University of Sheffield, Sheffield

[6] Wu A-M, Bisignano C, James SL et al (2021) Global, regional, and national burden of bone fractures in 204 countries and territories, 1990–2019: a systematic analysis from the Global Burden of Disease Study 2019. Lancet Healthy Longev 2:e580–e592 34723233 10.1016/S2666-7568(21)00172-0 PMC8547262

[7] Mafi Golchin M, Heidari L, Ghaderian SMH, Akhavan-Niaki H (2016) Osteoporosis: a silent disease with complex genetic contribution. J Genet Genomics 43:49–61 26924688 10.1016/j.jgg.2015.12.001

[8] Kanis JA, Oden A, Johansson H et al (2009) FRAX® and its applications to clinical practice. Bone 44:734–743 19195497 10.1016/j.bone.2009.01.373

[9] Kanis JA, Hans D, Cooper C et al (2011) Interpretation and use of FRAX in clinical practice. Osteoporos Int 22:2395 21779818 10.1007/s00198-011-1713-z

[10] Lee M-T, Fu S-H, Hsu C-C et al (2023) Epidemiology and clinical impact of osteoporosis in Taiwan: a 12-year trend of a nationwide population-based study. J Formos Med Assoc 122:S21–S35 37208247 10.1016/j.jfma.2023.05.001

[11] Tai T-W, Chen H-Y, Shih C-A et al (2024) Asia-Pacific consensus on long-term and sequential therapy for osteoporosis. Osteoporos Sarcopenia 10:3–10 38690538 10.1016/j.afos.2024.02.001 PMC11056428

[12] Kanis JA, Johansson H, Harvey NC, McCloskey EV (2018) A brief history of FRAX. Arch Osteoporos 13:118 30382424 10.1007/s11657-018-0510-0 PMC6290984

[13] Vandenput L, Johansson H, McCloskey EV et al (2022) Update of the fracture risk prediction tool FRAX: a systematic review of potential cohorts and analysis plan. Osteoporos Int 33:2103–2136 35639106 10.1007/s00198-022-06435-6

[14] Kanis JA, Harvey NC, Johansson H et al (2020) A decade of FRAX: how has it changed the management of osteoporosis? Aging Clin Exp Res 32:187–196 32043227 10.1007/s40520-019-01432-y

[15] Kanis JA, Harvey NC, Cooper C et al (2016) A systematic review of intervention thresholds based on FRAX: a report prepared for the National Osteoporosis Guideline Group and the International Osteoporosis Foundation. Arch Osteoporos 11:25–25 27465509 10.1007/s11657-016-0278-z PMC4978487

[16] Chandran M, Brind’Amour K, Fujiwara S et al (2023) Prevalence of osteoporosis and incidence of related fractures in developed economies in the Asia Pacific region: a systematic review. Osteoporos Int 34:1037–1053 36735053 10.1007/s00198-022-06657-8 PMC10202996

[17] Liu I-T, Liang F-W, Li C-C et al (2022) Validation of the Taiwan FRAX® calculator for the prediction of fracture risk. Arch Osteoporos 17:27 35094177 10.1007/s11657-022-01068-y

[18] Chang C-S, Chang Y-F, Liu P-Y et al (2012) Smoking, habitual tea drinking and metabolic syndrome in elderly men living in rural community: the Tianliao old people (TOP) study 02. PLoS ONE 7:e38874 22719971 10.1371/journal.pone.0038874 PMC3375307

[19] Chang C-S, Chang Y-F, Wang M-W et al (2013) Inverse relationship between central obesity and osteoporosis in osteoporotic drug naive elderly females: the Tianliao old people (TOP) study. J Clin Densitom 16:204–211 22717906 10.1016/j.jocd.2012.03.008

[20] Ou L-C, Sun Z-J, Chang Y-F et al (2013) Epidemiological survey of quantitative ultrasound in risk assessment of falls in middle-aged and elderly people. PLoS ONE 8:e71053 23951077 10.1371/journal.pone.0071053 PMC3737261

[21] Chao Y-J, Chen C-Y, Wu C-F et al (2010) The prevalence of osteoporosis and associated risk factors in elderly females living in a rural community in Taiwan. Taiwan J Family Med 20:64–73

[22] Wu CH, Chen HY, Chang YF, Chang CS, Chen CY, Yang YC et al (2011) Osteoporosis and status of management in old males lived in rural community in Taiwan. Osteoporos Int 22(Suppl 1):S314

[23] Li C-C, Ou L-C, Chang Y-F et al (2025) Performance and interventional cutoffs of osteoporosis self-assessment tools in the community: implications for screening and early referral. Osteoporos Int 36:2149–2155 40833502 10.1007/s00198-025-07656-1

[24] Liu I-T, Liang F-W, Wang S-T et al (2021) The effects of falls on the prediction of osteoporotic fractures: epidemiological cohort study. Arch Osteoporos 16:110 34245374 10.1007/s11657-021-00977-8

[25] Li C-C, Liu IT, Cheng T-T et al (2024) Decomposing and simplifying the Fracture Risk Assessment Tool-a module from the Taiwan-specific calculator. JBMR Plus 8:ziae039 38644977 10.1093/jbmrpl/ziae039 PMC11032218

[26] Writing Group for the ISCD Position Development Conference (2004) Position statement: executive summary. The Writing Group for the International Society for Clinical Densitometry (ISCD) Position Development Conference. J Clin Densitom 7:7–12 14742882 10.1385/jcd:7:1:7

[27] Tai T-W, Huang C-F, Huang H-K et al (2023) Clinical practice guidelines for the prevention and treatment of osteoporosis in Taiwan: 2022 update. J Formos Med Assoc 122:S4–S13 36781371 10.1016/j.jfma.2023.01.007

[28] Shao C-J, Hsieh Y-H, Tsai C-H, Lai K-A (2009) A nationwide seven-year trend of hip fractures in the elderly population of Taiwan. Bone 44:125–129 18848656 10.1016/j.bone.2008.09.004

[29] National Health Insurance Administration (2024) National health insurance annual report 2024–2025. National Health Insurance Administration, Taipei

[30] Fu S-H, Yu P-Y, Li C-Y et al (2023) Diagnostic accuracy of algorithms to define incident and second hip fractures: a Taiwan validation study. J Formos Med Assoc 122(Suppl 1):S82-s91 37353444 10.1016/j.jfma.2023.05.037

[31] Fine JP, Gray RJ (1999) A proportional hazards model for the subdistribution of a competing risk. J Am Stat Assoc 94:496–509

[32] Hsieh C-Y, Su C-C, Shao S-C et al (2019) Taiwan’s National Health Insurance Research Database: past and future. Clin Epidemiol 11:349–358 31118821 10.2147/CLEP.S196293 PMC6509937

[33] Richards C, Stevens R, Lix LM et al (2024) Fracture prediction in rheumatoid arthritis: validation of FRAX with bone mineral density for incident major osteoporotic fractures. Rheumatology (Oxford) 64:228–234 10.1093/rheumatology/kead676 38092036

[34] Adami G, Biffi A, Porcu G et al (2023) A systematic review on the performance of fracture risk assessment tools: FRAX, DeFRA, FRA-HS. J Endocrinol Invest 46:2287–2297 37031450 10.1007/s40618-023-02082-8 PMC10558377

[35] Allbritton-King JD, Elrod JK, Rosenberg PS, Bhattacharyya T (2022) Reverse engineering the FRAX algorithm: clinical insights and systematic analysis of fracture risk. Bone 159:116376 35240349 10.1016/j.bone.2022.116376 PMC9035136

[36] Leslie WD, Morin SN, Lix LM et al (2021) Fracture prediction from FRAX for Canadian ethnic groups: a registry-based cohort study. Osteoporos Int 32:113–122 32809043 10.1007/s00198-020-05594-8 PMC7611612

[37] Wu Q, Jung J (2025) Uncovering racial and genetic disparities in FRAX performance for fracture risk assessment in postmenopausal women. J Bone Miner Res. 10.1093/jbmr/zjaf160 41165774 10.1093/jbmr/zjaf160 PMC13034674

[38] Fuleihan GE-H, Chakhtoura M, Cauley JA, Chamoun N (2017) Worldwide fracture prediction. J Clin Densitom 20:397–424 28734709 10.1016/j.jocd.2017.06.008

[39] Kanis JA, Cooper C, Dawson-Hughes B et al (2020) FRAX and ethnicity. Osteoporos Int 31:2063–2067 32888046 10.1007/s00198-020-05631-6 PMC7116478

[40] Kanis JA, McCloskey EV, Johansson H et al (2010) Development and use of FRAX® in osteoporosis. Osteoporos Int 21:407–413 10.1007/s00198-010-1253-y 20464374

[41] Cosman F, de Beur SJ, LeBoff MS et al (2014) Clinician’s guide to prevention and treatment of osteoporosis. Osteoporos Int 25:2359–2381 25182228 10.1007/s00198-014-2794-2 PMC4176573

[42] Chan D-C, McCloskey EV, Chang C-B et al (2017) Establishing and evaluating FRAX® probability thresholds in Taiwan. J Formos Med Assoc 116:161–168 27117886 10.1016/j.jfma.2016.03.006

