# Supplementary material for: Ten-year re-validation of the fracture risk assessment tool (FRAX®) in Taiwan
Source: Arch Osteoporos. 2026 Jul 21;21(1):102. doi: 10.1007/s11657-026-01745-2 (PMC13388683; doi:10.1007/s11657-026-01745-2)
Supplement: Supplementary file 1 — Supplementary file1 (DOCX 202 KB) [file 11657_2026_1745_MOESM1_ESM.docx]

Supplemental Table S1. ICD-9-CM and ICD-10-CM inpatient diagnosis codes

|  | **ICD-9** | **ICD-10** |
| --- | --- | --- |
| Osteoporosis | 733.0 Osteoporosis  733.1 Pathologic fracture | M80 Osteoporosis with current pathological fracture  M81 Osteoporosis without current pathological fracture |
| Hip Fracture* | 733.14 Pathologic fracture of neck of femur  820 Fracture of neck of femur | S72 Fracture of femur |
| Vertebra Fracture | 733.13 Pathologic fracture of vertebrae  805.2 Closed fracture of dorsal [thoracic] vertebra without mention of spinal cord injury  805.3 Open fracture of dorsal [thoracic] vertebra without mention of spinal cord injury  805.4 Closed fracture of lumbar vertebra without mention of spinal cord injury  805.5 Open fracture of lumbar vertebra without mention of spinal cord injury  805.6 Closed fracture of sacrum and coccyx without mention of spinal cord injury  805.7 Open fracture of sacrum and coccyx without mention of spinal cord injury  805.8 Closed fracture of unspecified vertebral column without mention of spinal cord injury  805.9 Open fracture of unspecified vertebral column without mention of spinal cord injury | S22.0 Fracture of thoracic vertebra  S22.1  S32.0 Fracture of lumbar vertebra  S32.1 Fracture of sacrum  S32.2 Fracture of coccyx |
| Forearm Fracture | 733.12 Pathologic fracture of distal radius and ulna  813 Fracture of radius and ulna | S52 Fracture of forearm |
| Shoulder Fracture | 733.11 Pathologic fracture of humerus  812 Fracture of scapula  818 Ill-defined fractures of upper limb | S42.2 Fracture of upper end of humerus  S42.3 Fracture of shaft of humerus  S42.4 Fracture of lower end of humerus |

* Hip fracture ascertainment followed the validated inpatient algorithm described by Fu et al. [29]. Comparable validation studies are currently unavailable for non-hip fractures.

Supplemental Table S2. Calibration of FRAX-predicted 10-year probabilities of MOF and HF by age strata

|  | <65 years old | | >= 65 years old | |
| --- | --- | --- | --- | --- |
|  | O/E | 95% CI | O/E | 95% CI |
| FRAX with BMD |  |  |  |  |
| MOF | 1.04 | 0.73-1.43 | 1.23 | 1.06-1.42* |
| HF | 0.33 | 0.05-1.08 | 1.40 | 1.14-1.72* |
| FRAX without BMD |  |  |  |  |
| MOF | 0.81 | 0.58-1.12 | 1.16 | 1.00-1.34* |
| HF | 0.18 | 0.03-0.58** | 1.24 | 1.00-1.51* |

FRAX, fracture risk assessment tool; BMD, bone mineral density; MOF, major osteoporotic fracture; HF, hip fracture. * *p* < 0.05.

Supplemental Table S3. Calibration pattern across risk quintiles

| Quintile | O/E ratio* |  |  |  |
| --- | --- | --- | --- | --- |
|  | MOF_without_BMD | MOF_with_BMD | HF_without_BMD | HF_with_BMD* |
| 1 | 0.40(0.14-0.66) | 0.45(0.19-0.71) | Not estimable** | Not estimable |
| 2 | 0.78(0.46-1.10) | 0.65(0.37-0.94) | 0.31(0.00-0.61) | 0.09(0.00-0.27) |
| 3 | 1.09(0.75-1.43) | 0.99(0.68-1.31) | 1.14(0.58-1.66) | 0.85(0.38-1.32) |
| 4 | 1.28(0.96-1.59) | 1.52(1.18-1.86) | 1.17(0.69-1.67) | 1.90(1.28-2.53) |
| 5 | 1.06(0.87-1.25) | 1.02(0.83-1.21) | 1.46(1.09-1.82) | 1.21(0.89-1.53) |

* Confidence intervals for O/E ratio (Observed-to-Expected ratio) s were calculated using Poisson-based methods. MOF, major osteoporotic fracture. HF, hip fracture.

** O/E ratios were not estimable in the lowest quintile for hip fracture because no fracture events occurred in these subgroups.

Supplemental Table S4. Cox proportional hazard model with competing risk of MOF and HF for FRAX with BMD

| FRAX CRFs | MOF |  | HF |  |
| --- | --- | --- | --- | --- |
|  | Sub-distribution HR | *p* value | Sub-distribution HR | *p* value |
| Female | 1.59(1.08-2.33) | 0.0190 | 1.04(0.56-1.92) | 0.9018 |
| Age, year | 1.05(1.03-1.06) | <.0001 | 1.12(1.09-1.14) | <.0001 |
| BMI, kg/m^2^ | 1.00(0.97-1.04) | 0.9271 | 0.99(0.93-1.05) | 0.6429 |
| Previous Fracture | 1.83(1.28-2.61) | 0.0010 | 1.90(1.09-3.32) | 0.0235 |
| Parent Fractured Hip | 0.88(0.46-1.67) | 0.6917 | 0.97(0.31-3.00) | 0.9586 |
| Current Smoking | 1.57(0.97-2.56) | 0.0689 | 1.97(1.02-3.82) | 0.0452 |
| Alcohol | 1.39(0.77-2.50) | 0.2714 | 1.55(0.66-3.64) | 0.3140 |
| Glucocorticoids | 1.14(0.53-2.47) | 0.7336 | 2.09(0.78-5.59) | 0.1432 |
| Rheumatoid arthritis history | 2.17(0.79-5.98) | 0.1352 | 3.93(0.98-15.71) | 0.0527 |
| Secondary osteoporosis | 1.10(0.54-2.24) | 0.7863 | 1.04(0.31-3.49) | 0.9480 |
| Femoral neck T-score, SD | 0.67(0.59-0.77) | <.0001 | 0.66(0.53-0.81) | 0.0001 |

FRAX, fracture risk assessment tool; MOF, major osteoporotic fracture; HF, hip fracture; BMD, bone mineral density; CRFs, clinical risk factors; HR, hazard ratio; BMI, body mass index.

Supplemental Table S5. Cox proportional hazard model with competing risk of MOF and HF for FRAX without BMD in AOMs users

| FRAX CRFs | MOF |  | HF |  |
| --- | --- | --- | --- | --- |
|  | Sub-distribution HR | *p* value | Sub-distribution HR | *p* value |
| Female | 1.80(0.91-3.60) | 0.0938 | 1.08(0.43-2.72) | 0.8639 |
| Age, year | 1.06(1.01-1.10) | 0.0080 | 1.11(1.05-1.17) | 0.0003 |
| BMI, kg/m^2^ | 0.99(0.93-1.06) | 0.7979 | 0.99(0.89-1.10) | 0.8442 |
| Previous Fracture | 1.42(0.81-2.50) | 0.2222 | 1.38(0.57-3.32) | 0.4722 |
| Parent Fractured Hip | 0.96(0.34-2.69) | 0.9324 | 1.39(0.34-5.63) | 0.6451 |
| Current Smoking | 1.35(0.64-2.84) | 0.4342 | 1.12(0.38-3.31) | 0.8365 |
| Alcohol | 1.63(0.83-3.22) | 0.1577 | 1.32(0.31-5.63) | 0.7084 |
| Glucocorticoids | 0.82(0.30-2.24) | 0.6937 | 1.87(0.66-5.31) | 0.2421 |
| Secondary osteoporosis | 3.05(1.89-4.93) | <.0001 | 1.45(0.18-11.66) | 0.7265 |

FRAX, fracture risk assessment tool; MOF, major osteoporotic fracture; HF, hip fracture; AOMs, anti-osteoporosis medications; CRFs, clinical risk factors; HR, hazard ratio; BMI, body mass index.

Supplemental Table S6. Cox proportional hazard model with competing risk of MOF and HF for FRAX without BMD in non-AOMs users

| FRAX CRFs | MOF |  | HF |  |
| --- | --- | --- | --- | --- |
|  | Sub-distribution HR | *p* value | Sub-distribution HR | *p* value |
| Female | 1.47(0.98-2.22) | 0.0638 | 1.04(0.55-1.97) | 0.8946 |
| Age, year | 1.04(1.03-1.06) | <.0001 | 1.12(1.09-1.15) | <.0001 |
| BMI, kg/m^2^ | 0.96(0.91-1.01) | 0.0934 | 0.91(0.84-0.99) | 0.0306 |
| Previous Fracture | 1.71(1.06-2.76) | 0.0290 | 1.40(0.59-3.33) | 0.4510 |
| Parent Fractured Hip | 0.73(0.30-1.78) | 0.4937 | Not estimable* |  |
| Current Smoking | 1.11(0.62-1.97) | 0.7286 | 1.45(0.66-3.19) | 0.3588 |
| Alcohol | 1.06(0.53-2.13) | 0.8639 | 1.10(0.39-3.13) | 0.8560 |
| Glucocorticoids | 0.63(0.17-2.36) | 0.4932 | 0.79(0.10-6.33) | 0.8251 |
| Rheumatoid arthritis history | 3.35(1.22-9.22) | 0.0190 | 6.48(1.50-28.05) | 0.0124 |
| Secondary osteoporosis | 0.95(0.37-2.42) | 0.9150 | 1.14(0.27-4.86) | 0.8566 |

FRAX, fracture risk assessment tool; MOF, major osteoporotic fracture; HF, hip fracture; AOMs, anti-osteoporosis medications; BMD, bone mineral density; CRFs, clinical risk factors; HR, hazard ratio; BMI, body mass index.

* Estimate unstable because of sparse events.

Supplemental Table S7. The FRAX with BMD threshold for predicting MOF and HF

| MOF | | | | HF | | | |
| --- | --- | --- | --- | --- | --- | --- | --- |
| Threshold (%) | Sensitivity | Specificity | Youden | Threshold (%) | Sensitivity | Specificity | Youden |
| 20.0 ^a^ | 0.22 | 0.94 | 0.16 | 12.0 | 0.20 | 0.96 | 0.16 |
| 15.0 ^c^ | 0.37 | 0.85 | 0.22 | 11.0 | 0.24 | 0.95 | 0.19 |
| 12.0 | 0.52 | 0.77 | 0.29 | 10.0 | 0.27 | 0.94 | 0.21 |
| 11.0 | 0.55 | 0.74 | 0.29 | 9.0 | 0.31 | 0.92 | 0.23 |
| 10.0 | 0.60 | 0.72 | 0.31 | 8.0 | 0.34 | 0.89 | 0.24 |
| 9.5 ^b^ | 0.64 | 0.70 | 0.33 | 7.0 ^c^ | 0.40 | 0.86 | 0.26 |
| 9.0 | 0.66 | 0.67 | 0.33 | 6.0 ^c^ | 0.47 | 0.83 | 0.30 |
| 8.5 | 0.70 | 0.65 | 0.35 | 5.0 | 0.65 | 0.78 | 0.43 |
| 8.2 ^d^ | 0.72 | 0.63 | 0.35 | 4.5 | 0.72 | 0.75 | 0.47 |
| 8.0 | 0.73 | 0.62 | 0.35 | 4.0 ^b^ | 0.77 | 0.71 | 0.48 |
| 7.5 | 0.77 | 0.58 | 0.34 | 3.8 ^d^ | 0.82 | 0.69 | 0.51 |
| 7.0 | 0.78 | 0.54 | 0.32 | 3.5 | 0.83 | 0.66 | 0.49 |
| 6.5 | 0.80 | 0.51 | 0.31 | 3.0 ^a^ | 0.86 | 0.62 | 0.48 |
| 6.0 | 0.84 | 0.47 | 0.31 | 2.0 | 0.94 | 0.51 | 0.44 |
| 5.0 | 0.89 | 0.38 | 0.28 | 1.0 | 1.00 | 0.34 | 0.34 |

FRAX, fracture risk assessment tool; BMD, bone mineral density; MOF, major osteoporotic fracture; HF, hip fracture.

a. NOF threshold [39, 40], b. Liu’s threshold [17], c. Chan’s threshold [41], d. this study suggested threshold with highest Youden index.

Supplemental Figure S1. The FRAX with BMD threshold for predicting major osteoporotic fracture


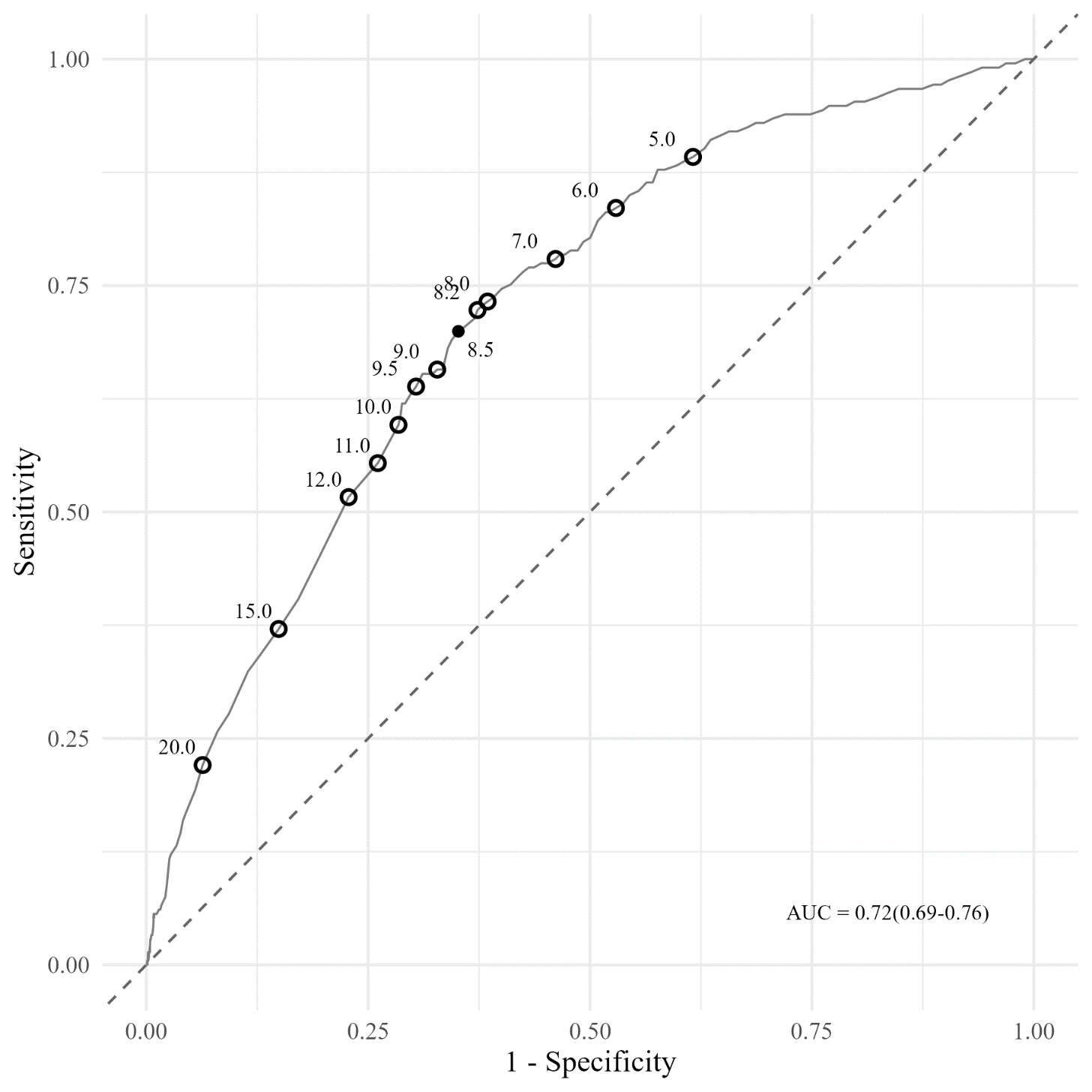


The gray lines depict the raw ROC curves calculated from the observed sensitivity and specificity values. Hollow circles indicate pre-defined threshold positions. A corresponding threshold label accompanies each marker. A solid circle (threshold = 8.5) indicates the clinically recommended threshold.

Supplemental Figure S2. The FRAX with BMD threshold for predicting hip fracture


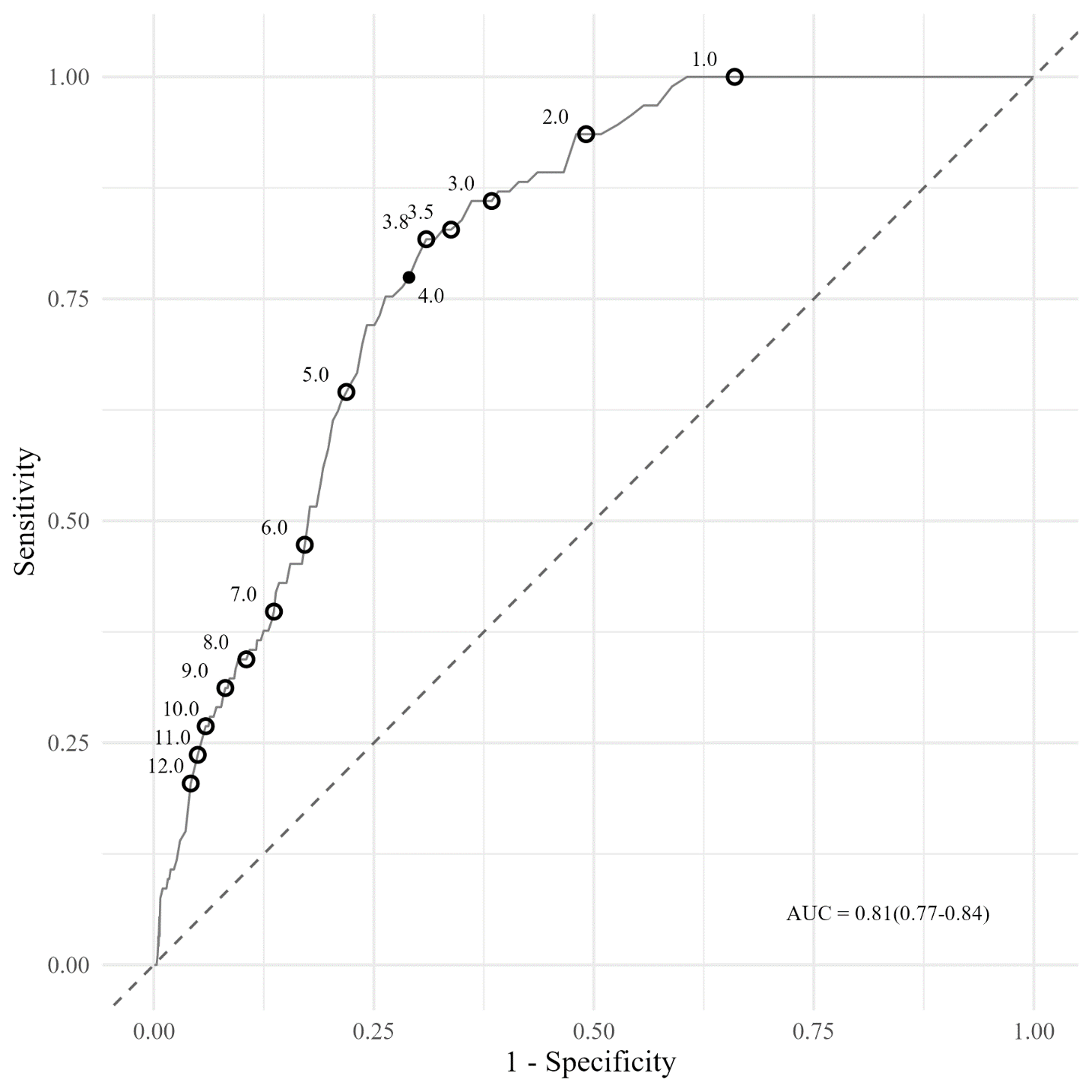


The gray lines depict the raw ROC curves calculated from the observed sensitivity and specificity values. Hollow circles indicate pre-defined threshold positions. A corresponding threshold label accompanies each marker. A solid circle (threshold = 4.0) indicates the clinically recommended threshold.
